# Differential Effects of Serotonin Transporter Genotype on Anxiety-Like Behavior and Cognitive Judgment Bias in Mice

**DOI:** 10.3389/fnbeh.2019.00263

**Published:** 2019-12-03

**Authors:** Viktoria Krakenberg, Vanessa Tabea von Kortzfleisch, Sylvia Kaiser, Norbert Sachser, S. Helene Richter

**Affiliations:** ^1^Department of Behavioural Biology, University of Münster, Münster, Germany; ^2^Otto Creutzfeldt Center for Cognitive and Behavioral Neuroscience, University of Münster, Münster, Germany

**Keywords:** anxiety, serotonin transporter, 5-HTT, cognitive judgment bias, pessimism, endophenotype, mice

## Abstract

In humans, the short allele of a common polymorphism in the serotonin transporter (5-HTT) gene is associated with a higher risk to develop depression and anxiety disorders. Furthermore, individuals carrying this allele are characterized by negative judgment biases, as they tend to interpret ambiguous information in a more pessimistic way. 5-HTT knockout mice, lacking the 5-HTT gene either homo- or heterozygously, provide a widely used model organism for the study of symptoms related to human anxiety disorders. In the present study, we aimed to prove the anxiety-like phenotype of the 5-HTT mouse model, and to investigate whether 5-HTT genotype also causes differences in judgment bias. While our results confirm that homozygous 5-HTT knockout mice display highest levels of anxiety-like behavior, it was decreased in heterozygous mice. Against our expectations, we did not detect differences in the animals’ judgment bias. These results indicate that at least in mice the association between 5-HTT genotype and judgment bias is not straightforward and that other factors, including multiple genes as well as environmental influences, are implicated in the modulation of judgment biases. More research is needed to gain further insights into their function as potential endophenotypes for psychopathology.

## Introduction

Anxiety disorders are among the most prevalent psychiatric disorders, severely compromising an individual’s quality of life (Bandelow and Michaelis, 2015). Environmental and genetic factors as well as their interaction are regarded as contributing causes (Caspi and Moffitt, 2006; Caspi et al., 2010). Among the genetic risk factors, the serotonin transporter (5-HTT) gene constitutes an important candidate, with a common polymorphism in the 5-HTT-linked polymorphic region, appearing in form of either a short (s) or a long (l) allele (Lesch et al., 1996; Gross and Hen, 2004; Canli and Lesch, 2007). Carrying the s-allele has been linked to anxiety-related personality traits and is associated with an increased risk to develop anxiety disorders and depression, especially following adverse life events (Lesch et al., 1996; Mazzanti et al., 1998; Greenberg et al., 2000; Caspi et al., 2003, 2010).

A shared characteristic of individuals with anxiety disorders is that they selectively process information in a way favoring negative emotional valence, a phenomenon also referred to as *cognitive bias* (Mathews and MacLeod, 1994, 2005). Such negative cognitive biases comprise *attention* (e.g., enhanced attention toward threatening stimuli), *memory* (e.g., the predominant recall of negative memories), and *judgment biases* (e.g., pessimistic interpretations of ambiguous information) (Mathews and MacLeod, 2005). Evidence suggests a genetic component of such biases, with the 5-HTT polymorphism being implicated in their development and maintenance (Fox et al., 2009; Fox and Standage, 2012). For instance, 5-HTT s-allele carriers were found to display enhanced attention toward and no active avoidance of negative information (Beevers et al., 2007; Fox et al., 2009), and to interpret ambiguous homophones more pessimistically compared to l-allele carriers (Fox and Standage, 2012). Therefore, cognitive biases may provide important endophenotypes for anxiety disorders (Fox et al., 2009; Fox and Standage, 2012).

Based on the human cognitive bias concept, the development of the first cognitive bias test for rats (Harding et al., 2004) paved the way for the assessment of judgment biases in a variety of animal species (e.g., Kloke et al., 2014; Bethell, 2015; Hintze et al., 2018; Jones et al., 2018; Krakenberg et al., 2019). In animals, similar to humans, negative judgment biases often co-occur with higher levels of anxiety-like and depression-related behavior, implying that the underlying mechanisms might be shared among species (but see for instance Bethell and Koyama, 2015). This was mainly shown on the basis of studies using emotion-manipulating treatments to induce specific emotional states. For instance, isolation stress in chicken (Salmeto et al., 2011), as well as changes in light intensity or chronic stress exposure in rats (Burman et al., 2009; Rygula et al., 2013) were reflected in the animals’ judgment bias. Likewise, also approaches using genetic animal models without previous induction of anxiety- or depression-like states revealed mood-congruent differences in judgment bias (e.g., congenitally helpless rat strains) (Enkel et al., 2010; Richter et al., 2012; Kloke et al., 2014).

As mice are the predominantly used mammalian animal model, the aim of the present study was to investigate, whether cognitive biases may constitute potential endophenotypes for anxiety disorders in this species. Therefore, we applied the 5-HTT mouse model, a well-established model for the study of human anxiety disorders (Bengel et al., 1998). While 5-HTT wild type mice (+/+) express the normal amount of 5-HTT, its expression rate is reduced by 50% in heterozygous knockout mice (+/−) and 5-HTT is completely absent in homozygous knockout mice (−/−) (Bengel et al., 1998; Murphy et al., 2001). 5-HTT −/− mice are characterized by a robust increase in anxiety-like behavior, as well as a decrease in exploratory locomotion (Holmes et al., 2003; Carroll et al., 2007; Heiming et al., 2009), while 5-HTT +/− mice only display these behavioral traits when they have experienced experimentally induced adversity (Carola et al., 2008; Jansen et al., 2010; van den Hove et al., 2011). Furthermore, there is a first hint for a link between negative cognitive biases and 5-HTT deficiency in the mouse model, as Kloke et al. (2014) found 5-HTT −/− mice to display a trend for a pessimistic-like judgment bias in a spatial judgment bias paradigm.

To further investigate the role of judgment biases as potential endophenotypes for anxiety disorders, we tested mice varying in 5-HTT genotype in both a judgment bias paradigm and a battery of standardized tests for anxiety-like behavior. More precisely, judgment bias assessment was conducted using a recently implemented, automated touchscreen paradigm (Krakenberg et al., 2019). This refined method increases the translational value of the task, while at the same time offers automation-related advantages such as a reduced experimenter effect and highly accurate data acquisition (Bussey et al., 2012; Talpos and Steckler, 2013; Krakenberg et al., 2019). Against the background elucidated above, we hypothesized that mice of the three genotypes would differ in their cognitive judgment bias (CJB). We further expected a negative judgment bias to co-occur with higher levels of anxiety-like behavior.

## Animals and Methods

### Animals and Housing Conditions

The study was conducted using a serotonin transporter (5-HTT) knockout mouse model (Bengel et al., 1998). We studied male wild type (+/+; *n* = 15), heterozygous (+/−; *n* = 14) and homozygous (−/−; *n* = 11) knockout mice (deviations from sample sizes due to technical problems during behavioral testing are indicated in the results section). As six mice (+/+: *n* = 1; +/−: *n* = 2; −/−: *n* = 3) displayed a continuous lack of improvement in training performance they were excluded from the study and are not included in the above mentioned sample sizes. The animals originated from the internal breeding stock of the Department of Behavioral Biology at the University of Münster, Germany. The original heterozygous breeding pairs were provided by the Department of Molecular Psychiatry at the University of Würzburg, Germany. For genotyping, genomic DNA was extracted from ear tissue and amplified by PCR. Genotypes were identified by agarose gel electrophoresis of DNA fragments with a lengths of 225 bp (5-HTT +/+), 272 bp (5-HTT −/−) or both (5-HTT +/−). After weaning, mice were housed in groups of 2–5 animals per cage (Makrolon cages type III, 38 × 23 × 15 cm^3^). At the age of approximately 9 weeks, they were transferred to single cages to avoid any escalated aggression. For four mice (+/+: *n* = 1;+/−: *n* = 2; −/−: *n* = 1), single housing had to be initiated before that age due to incidences of escalated fighting. Cages contained wood shavings as bedding material (Allspan, Höveler GmbH & Co. KG, Langenfeld, Germany), a paper towel, a wooden stick and a semi-transparent red plastic house (11.1 × 11.1 × 5.5 cm^3^, Tecniplast Deutschland GmbH, Hohenpeißenberg, Germany). Housing rooms were maintained at a reversed dark/light cycle with lights off at 10.00 a.m., a temperature of approximately 22°C, and a relative humidity of about 50%. Mice were provided with water and food (Altromin 1314; Altromin Spezialfutter GmbH & Co. KG, Lage, Germany) *ad libitum*, except for the time during touchscreen training and cognitive bias testing, when they were food restricted to 90–95% of their *ad libitum* feeding weights in order to enhance their motivation to work for food rewards. During the food restriction period, the animals’ body weights remained constant. For details regarding the animals’ body weights see Supplementary Figure S4.

### Ethics Statement

All procedures complied with the regulations covering animal experimentation within Germany (Animal Welfare Act) and the EU (European Communities Council DIRECTIVE 2010/63/EU) and were approved by the local (Gesundheits-und Veterinäramt Münster, Nordrhein-Westfalen) and federal authorities (Landesamt für Natur, Umwelt und Verbraucherschutz Nordrhein-Westfalen “LANUV NRW,” reference number 84-02.04.2015.A441).

### Experimental Design

**FIGURE 1:**
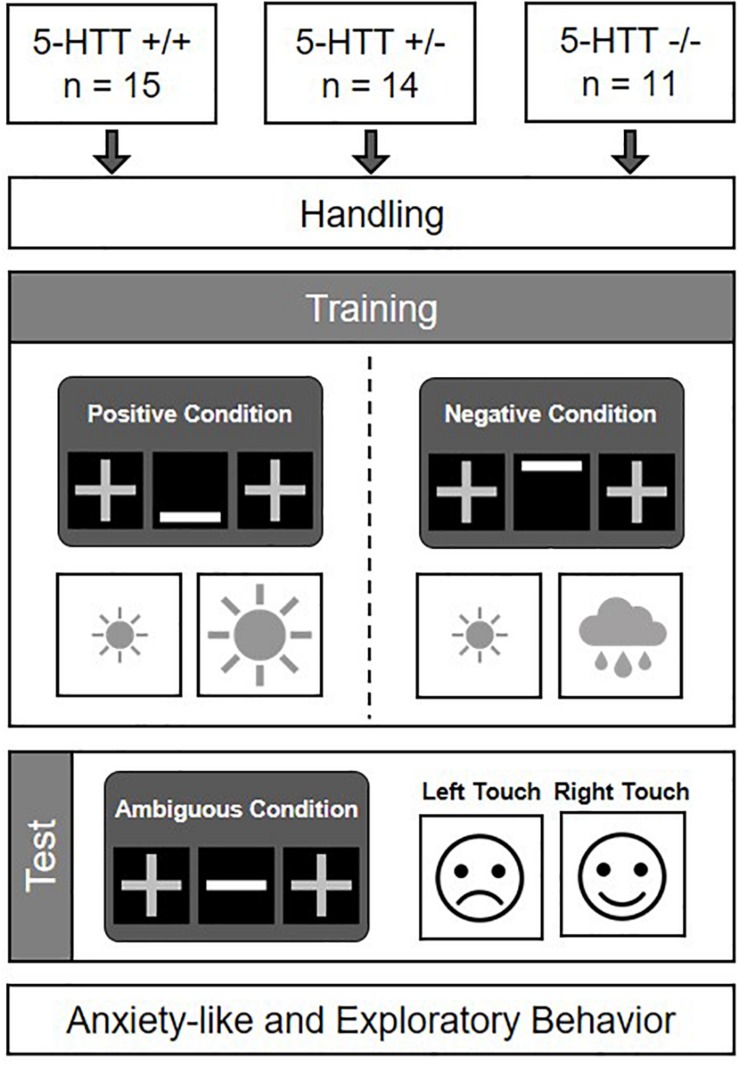
Experimental design. 5-HTT +/+, +/−, and −/− mice were accustomed to cup handling and afterwards trained in a discrimination task. Following successful discrimination learning, their cognitive judgment bias was assessed (cf. Krakenberg et al., 2019). Subsequently, they were tested in a battery of behavioral tests to assess their anxiety-like and exploratory behavior.

The aims of this study were (I) to assess CJB as a potential cognitive endophenotype for anxiety disorders in mice varying in their serotonin transporter genotype (5-HTT +/+, +/−, and −/−), and (II) to confirm the anxiety-like phenotype of the mouse model applied. The experiment comprised four phases: a handling phase, a training phase, a CJB test phase, and a behavioral test phase (see Figure 1). Mice entered the experimental phase batch-wise, with each batch comprising animals of at least two different genotypes. The handling phase started at the age of 10 ± 1 weeks. During this phase, mice were accustomed to cup handling, a method suggested to reduce anxiety in mice (Hurst and West, 2010). The individual weights of all mice were monitored on each experimental day until the start of the behavioral test phase. During the training phase, which started at 12 ± 1 weeks, mice had to acquire a discrimination task. As training success was determined on an individual basis, training durations differed among animals (for a comparison of training durations between groups see Supplementary Figure S1). After successful discrimination training, mice proceeded to the CJB test phase at an age of 30 ± 12 weeks. At the end of this phase, they were fed *ad libitum* diet again. 4 ± 1 weeks later, mice entered a battery of behavioral tests comprising the Elevated plus maze test (EPM), Dark light test (DL), Open field test (OF), and Free exploration test (FE), in order to assess their anxiety-like and exploratory behavior. The experimenter was blind to the genotypes of all mice during the experimental phase.

### Cognitive Judgment Bias Test

Cognitive judgment bias was assessed using an automated touchscreen system for mice (Bussey-Saksida Mouse Touch Screen Chambers, Model 80614, Campden Instruments Ltd., Loughborough, United Kingdom) according to the protocol established recently by our group with minor modifications (Krakenberg et al., 2019).

#### Apparatus

The touchscreen system has been described previously (Richter et al., 2014; Krakenberg et al., 2019). Briefly, it comprised four independent trapezoid shaped chambers (base area: 260 cm^2^, height: 20 cm), each equipped with a tone generator, a house light, an infrared sensitive touchscreen at the front (24.5 × 18.5 cm^2^) and a reward magazine (2 × 2 × 2 cm^3^) with a well for reward collection at the rear end. Rewards consisted of servings of sweet condensed milk, diluted 1:4 in tap water (in the following referred to as “SCM”). The touchscreen was covered by a black Perspex mask with three adjoining windows (7 × 7 cm^2^) providing access to the screen. On the central window (*cue presentation field*), cues in form of white bars (6 × 1 cm^2^) could be displayed at different positions. Mice had to nose poke a gray cross (width: 6 cm, height: 6 cm) on either the left or right window (*touch fields*) in response to these cues.

#### Paradigm

The paradigm was based on a discrimination task, in which mice had to learn to distinguish between a positive and a negative condition, signaled either by a bar displayed at the bottom (positive condition, P), or at the top of the cue presentation field (negative condition, N). During the positive condition, one touch field was associated with a large (12 μl SCM), the other one with a small reward (4 μl SCM). Mice had to learn to touch the high-rewarded field in this condition. During the negative condition, however, this previously high-rewarded touch field was associated with a mild “punishment” (5 s time out and house light on). The opposite field was again associated with a small reward (4 μl SCM). Mice had to learn to touch the small-rewarded field in this condition. While the cues signaling the positive or the negative condition, respectively, were the same for all mice, the association of cue (positive/negative) and correct touch field (left/right) was counterbalanced across animals of the three treatment groups. As soon as a mouse had acquired the task, it was tested in the CJB test. During the test, each animal was presented with cues displayed at three intermediate positions: middle (M), near positive (NP), and near negative (NN). The M condition was regarded as most ambiguous. The NP and NN conditions were also regarded as ambiguous, however, to a lesser degree. Responses to these ambiguous cues served as a measure of the animals’ judgment bias (Figure 1).

#### Procedure

Touchscreen training was carried out on a daily basis with exceptional breaks of about 3 days. For each touchscreen session, a mouse was taken out of its home cage, weighed, and transported to the adjacent room within a red, semi-transparent box (21 × 21 × 15 cm^3^). Consequently, the mouse was placed into a touchscreen chamber, the respective session was started and ended after a predetermined duration or as soon as a maximum number of trials was completed, depending on the respective training step. After completion of each touchscreen session, mice were returned to their home cages and received their daily food ration.

At the beginning of the training phase, mice acquired basic skills to operate within the chambers, such as touching for a reward, initiating trials by nose poking into the reward magazine and becoming accustomed to the mild “punishment” upon incorrect touches. This phase is commonly referred to as pre-training and followed the protocol described previously (Krakenberg et al., 2019).

Subsequently, mice entered the actual discrimination training. It was conducted according to our previously established protocol with some modifications (for details see following section and Krakenberg et al., 2019). The aim of this phase was the reliable discrimination between the positive and the negative condition by the mice. An overview of all discrimination training steps with final criteria for progression to the respective next training step is given in Table 1. The time of each training session was limited to 30 min.

**TABLE 1:** Discrimination training steps.

**Discrimination**	**Max. number**		**Correction**
**training**	**of trials**	**Criterion**	**trials**
1	50	≥5 sessions, completion of 50 trials in ≤20 min. in last two sessions	no
2	20	80% correct responses in two consecutive sessions	yes
3	50	80% correct responses in two consecutive sessions	yes
4	50	80% correct responses in one session	no
5	50	≥3 sessions, 80% correct responses in last two sessions	no

*Training consisted of 5 steps. Training sessions ended after 30 min, unless the maximum number of trials was reached before that time. Upon incorrect touches, correction trials (i.e., repeated presentation of the same trial until a correct touch) were presented (not included in the total trial count).*

Divergent from our previous protocol, we refined the first discrimination training step, which we hereafter refer to as discrimination training 1. This step had to be implemented during the ongoing experimental phase, as the direct transition to the consecutive step (discrimination training 2) resulted in substantial learning difficulties of the mice. During discrimination training 1, mice were presented with 25 positive and 25 negative trials per session in a pseudo-randomized order. In response to each cue, only touches on the correct touch field resulted in an outcome (large reward during positive condition, small reward during negative condition), while the respective incorrect touch field was inactivated so that touches on this field did not result in any outcome at all. A reasonable criterion for the progression to the next step turned out to be the completion of at least five sessions, with the last two sessions being completed in less than 20 min. This criterion was developed during the course of the experiment and was thus not applied to all animals equally. If animals did not show an improvement of learning performance during later training steps, they were returned to discrimination training 1.

Subsequently, mice proceeded to the regular training schedule, comprising discrimination trainings 2, 3, and 4. Briefly, they were confronted with balanced numbers of positive and negative trials in a pseudo-randomized order. Trial numbers increased from 20 (discrimination training 2) to 50 (discrimination trainings 3 and 4). Until discrimination training 3, incorrect touches were followed by correction trials, i.e., the same trial was repeatedly presented until a correct touch was executed. As soon as a response accuracy of 80% was reached, this was confirmed once more without correction trials in discrimination training 4.

We furthermore added another training step to the previous protocol, discrimination training 5, during which mice were confronted with so called “pseudo-probe trials.” These were pseudo-randomly chosen positive or negative training trials (3 × P and 3 × N per training session). Positive pseudo-probe trials remained unrewarded, regardless of whether mice touched correctly or incorrectly. Negative pseudo-probe trials remained unrewarded when touching correctly, but also unpunished when touching incorrectly. Firstly, this way of partial reinforcement was introduced in order to accustom the animals to the probe trials during the following test phase, which were neither rewarded nor punished. Secondly, these pseudo-probe trials were applied to prevent mice from learning the outcome of the ambiguous trials during testing (Roelofs et al., 2016). Per session, six pseudo-probe trials were presented and interspersed with 44 regular training trials. Mice had to stay in discrimination training 5 for a minimum of three sessions, and could proceed after the last two sessions were completed with at least 80% correct responses.

Once mice had successfully completed the discrimination task, they were tested for their CBJ. Subjects underwent five test sessions at intervals of approximately 24 h with maximally one gap day in between. For this purpose, mice were first weighed and then carried to the adjacent room in the red transport box, where the touchscreen session was started. Each test session comprised 50 trials. Per session, six probe trials (2 × NP, 2 × M, 2 × NN) were pseudo-randomly interspersed with 44 regular training trials (22 × P, 22 × N), amounting to a total number of 50 trials per test session. Touches in response to the ambiguous probe trials resulted in a neutral outcome, so no reward nor a “punishment” was presented. Instead, upon touching, cue as well as touch symbols disappeared and the animal could initiate the next trial. At the end of the test phase, each ambiguous cue had been presented 10 times, each trained cue 110 times, amounting to a total number of 250 trials that were completed during the test phase.

#### Behavioral Measures

The responses to the ambiguous cues during test sessions were taken as a measure of the animals’ CBJ. “Optimistic” choices were defined as touches according to the positive condition, “pessimistic” choices as touches according to the negative condition. Out of these choices in response to each condition, a score (*choice score*) was calculated as follows:

$$\mathrm{Choice}\,\mathrm{score}=\frac{N\,\mathrm{choices}\,\left(“\mathrm{optimistic}\,\text{”}\right)-N\,\mathrm{choices}\,\left(“\mathrm{pessimistic}\,\text{”}\right)}{N\,\mathrm{choices}\,\left(“\mathrm{optimistic}\,\text{”}+“\mathrm{pessimistic}\,\text{”}\right)}$$

Scores could range from −1 to +1. Higher choice scores indicate a higher proportion of “optimistic” choices, i.e., a more positive CBJ as compared to lower choice scores. Please note that the term “choice score” was chosen for the sake of intuitiveness. It is not supposed to imply that positive scores can be regarded as “optimistic” and negative scores as “pessimistic” *per se*. Scores should always be interpreted in relation to each other.

### Anxiety-Like and Exploratory Behavior

Mice were subjected to a battery of four behavioral tests, assessing state as well as trait anxiety and exploratory locomotion. State anxiety, defined as the anxiety an individual experiences at a specific moment in time and in response to an anxiogenic stimulus, was assessed in our study by conducting the EPM test, DL test, and OF test (Lister, 1990). Trait anxiety, on the contrary, is considered to be an enduring feature of an individual and was assessed using the FE test (Lister, 1990; Griebel et al., 1993; Ramos and Mormède, 1998). The test battery was scheduled 4 ± 1 weeks after the cognitive bias test at an age of 35 ± 12 (EPM, DL, and OF) and 36 ± 12 weeks (FE). To avoid any influence of food restriction on test outcomes, mice were fed *ad libitum* diet after CJB testing again. Tests were carried out at intervals of at least 48 h and at the beginning of the dark phase between 10.15 and 12.00 a.m. Before each test, the respective apparatus was cleaned with 70% ethanol. Mice were carried to the testing room within the red transport box for the EPM, DL, and OF and within their home cage for the FE. All tests were recorded with a camera (Logitech Webcam Pro 9000) and the video tracking software ANY-maze (version 4.99M, release: 2014, Stoelting Co., Wood Dale, IL, United States). Immediately after each test was started, the experimenter left the room quietly. The order of mice tested on the same day was randomized.

#### Elevated Plus Maze Test (EPM)

The apparatus of the EPM (Pellow et al., 1985; Lister, 1987, 1990) consisted of a plus-shaped maze, elevated 50 cm above the floor. Two opposing closed and open arms (30 × 5 cm^2^), respectively, extended from a central square area (5 × 5 cm^2^). Closed arms were surrounded by gray, 20 cm high wooden walls, open arms by a 4 mm high border to prevent the mice from falling down. The floor of the maze was covered by a gray PVC inlay. Illumination intensity in the central square was 25 lux. Mice stayed in the transport box for 1 min and were then placed on the central square of the EPM, always facing the same closed arm. They were allowed to explore the maze for 5 min. The relative time spent on the open arms, the relative number of entries into the open arms and the distance traveled on the open arms were taken as measures of the animals’ anxiety-like behavior. The sum of entries made into the open and closed arms of the apparatus was taken as a measure of their exploratory locomotion.

#### Dark Light Test (DL)

The apparatus of the DL test (Crawley and Goodwin, 1980) consisted of a modified Makrolon cage type III. The cage was divided into a light and a dark compartment by a gray PVC wall. The wall had an opening (10 × 4 cm) which could be closed with a sliding door. The dark compartment measured one third of the cage size, its walls were painted black and it could be closed with a gray PVC lid. The light compartment was illuminated with an intensity of 40 lux. Mice were placed into the dark compartment with the sliding door closed. After 1 min, the sliding door was opened and the mouse could freely explore the apparatus for 5 min. The time spent in and the latency to enter the light compartment were used as measures of the animals’ anxiety-like behavior, the number of entries made into the light compartment as a measure of their exploratory locomotion.

#### Open Field Test (OF)

The apparatus of the OF test (Archer, 1973; Treit and Fundytus, 1989) consisted of a white coated plywood box, comprising a square arena (80 × 80 cm^2^) framed by 42 cm high walls. Light intensity in the center of the arena was set to 35 lux. After arrival in the testing room, mice stayed in the transport box for 1 min before testing started. Mice were then placed into the arena, always facing the same corner, and were allowed to explore the apparatus for 5 min. Measures of anxiety-like behavior were the time spent in as well as the number of entries made into the center of the OF, defined as the area being located at least 20 cm distant from the walls. The total distance traveled was taken as a measure of exploratory locomotion.

#### Free Exploration Test (FE)

The apparatus of the FE test (Griebel et al., 1993) consisted of a white coated plywood box, comprising a square arena (60 × 60 cm^2^) framed by 35 cm high walls. In one wall there was an opening measuring 11 × 15 cm^2^. Light intensity in the center of the arena was set to 35 lux. The home cage of the animals was connected to the arena via a Plexiglas tunnel attached to the opening. The animals were transported into the testing room in their home cages, which were covered with black cloth to protect them from light. Mice were placed into the red transport box for 1 min after arrival in the testing room, before they were transferred to the home cage again. The home cage was equipped with a sliding door for this test, which was opened just before the start of the test and the animals could freely explore the arena for 15 min. The time spent in and the latency to enter the arena were used as measures of the animals’ anxiety-like behavior, the number of entries made into the arena as a measure of their exploratory locomotion.

### Statistics

To check for the assumptions of parametric analysis, residuals were examined for heteroscedasticity and normal distribution graphically and using the Shapiro–Wilk normality test. If the assumptions were not met, data were transformed using square root (DL: entries into light compartment, FE: latency to enter arena) or logarithmic transformations (OF: time spent in center and entries into center). CBJ test data were analyzed using a linear mixed-effects model (LMM) including “condition” as fixed within-subject factor, “genotype” as fixed between-subject factor and “age” as a numeric, random between-subject factor (lmer (score ∼ gen^∗^cond + (1| ID) + (1| age), data = data)). Behavioral test data were analyzed using LMM with “genotype” as fixed between-subject factor and “age” as numeric, random between-subject factor (lmer (a ∼ gen + (1| age), data = data)). Denominator degrees of freedom were rounded to the nearest integer. Tukey’s test was used for *post hoc* comparisons. Partial eta squared (η^2^_p_) was calculated to provide a standardized measure for the reported effects (Lakens, 2013). All statistical analyses were carried out using the software package “R project”^1^ [open source; packages: lme4 (Bates et al., 2015), lmerTest (Kuznetsova et al., 2017), lsmeans (Lenth, 2016)]. Differences were considered significant at *p* ≤ 0.05 and a trend at 0.05 < *p* ≤ 0.1.

## Results

### Cognitive Judgment Bias

**FIGURE 2:**
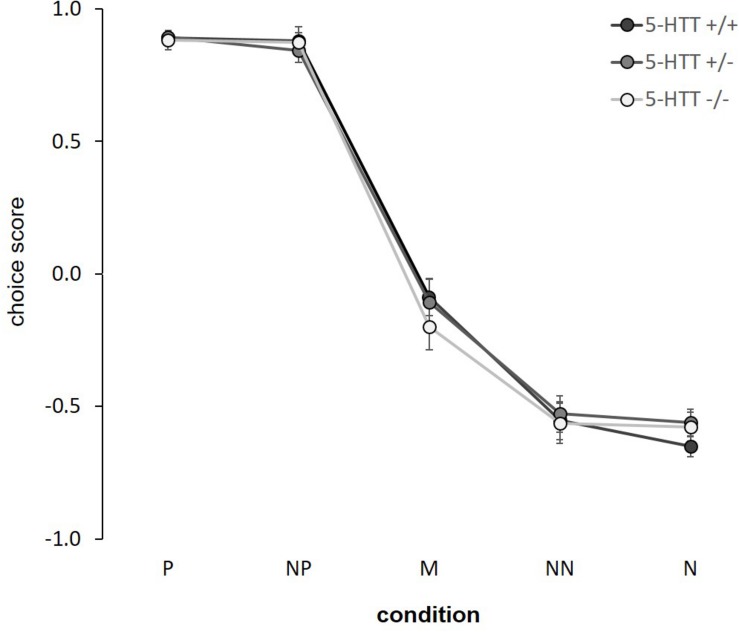
Cognitive judgment bias. Choice scores are given as means ± SEM. Statistics: LMM with “condition” as fixed within-subject factor, “genotype” as fixed between-subject factor and “age” as a random between-subject factor. Main effect of condition (*p* ≤ 0.001). Sample sizes: *n*+/+ = 15, *n*+/− = 14, *n*−/− = 11. P = positive, NP = near positive, M = middle, NN = near negative, N = negative.

There was a significant main effect of condition in the cognitive bias test (*F*_(__4_,_156__)_ = 501.725, *p* ≤ 0.001, η^2^_p_ = 0.93; Figure 2). *Post hoc* comparisons showed significant differences in the choice scores between adjacent conditions (*p* ≤ 0.001), except between P-NP and NN-N (*p* ≥ 0.8). On a descriptive level, choice scores resulted in a response curve, decreasing from the positive to the negative condition. However, we neither detected a main effect of genotype (*F*_(__2_,_83__)_ = 0.096, *p* = 0.909, η^2^_p_ = 0.002), nor a condition x genotype interaction (*F*_(__8_,_156__)_ = 0.459, *p* = 0.883, η^2^_p_ = 0.02). For statistical details and individual response curves see Supplementary Figure S3 and Supplementary Table S1.

### Anxiety-Like and Exploratory Behavior

Anxiety-like and exploratory behavior of the animals was assessed using the EPM, DL test, OF test, and FE test. While the animals’ behavior in the EPM, DL, and OF reflects their state anxiety, trait anxiety was assessed in the FE. An overview of statistical parameters of the analysis is given in Table 2. For graphs of all parameters assessed see Supplementary Figure S2.

**TABLE 2:** Statistical analysis of anxiety-like and exploratory behavior.

**Parameter**		**5-HTT +/+**	**5-HTT +/−**	**5-HTT −/−**	**ANOVA**		**Effect size**		**Tukey’s test, *p***		
							
		**Mean ± SEM**	**Mean ± SEM**	**Mean ± SEM**	***F***	***p***	**η2p**	**Trans**	**+/+ vs. +/−**	**+/+ vs. −/−**	**+/− vs. −/−**
**Elevated plus maze test**		***n* = 15**	***n* = 14**	***n* = 11**							
Time spent on open arm (rel.)	A	18.0 ± 2.7	30.0 ± 4.6	5.3 ± 1.7	38.562	**<0.001**	0.87	NT	**0.010**	**<0.001**	**<0.001**
Entries into open arms (rel.)	A	33.0 ± 2.5	37.0 ± 3.6	18.8 ± 4.1	8.404	**0.002**	0.41	NT	0.428	**0.023**	**0.002**
Distance traveled on open arms (m)	A	1.1 ± 0.2	2.2 ± 0.4	0.2 ± 0.1	25.384	**<0.001**	0.76	NT	**0.002**	**0.012**	**<0.001**
Sum of entries (#)	L	23.5 ± 1.6	26.1 ± 1.9	17.1 ± 2.0	7.255	**0.006**	0.47	NT	1.000	**0.013**	**0.016**
**Dark light test**											
Latency to enter light compartment (s)	A	6.7 ± 4.7	4.4 ± 1.0	10.5 ± 5.0	0.851	0.435	0.04	Log	0.755	0.406	0.813
Time spent in light compartment (s)	A	56.6 ± 10.3	52.6 ± 7.6	23.8 ± 11.3	2.859	***0.072***	0.15	NT	0.900	***0.073***	0.176
Entries into light compartment (#)	L	11.3 ± 1.8	11.3 ± 1.7	7.0 ± 2.4	1.817	0.178	0.10	SR	0.999	0.228	0.227
** Open field test**											
Time spent in center (s)	A	10.8 ± 2.2	11.2 ± 2.0	3.1 ± 1.7	11.380	**<0.001**	0.39	Log	0.977	**<0.001**	**<0.001**
Entries into center (#)	A	7.1 ± 1.4	8.1 ± 1.1	1.6 ± 0.8	14.710	**<0.001**	0.45	Log	0.673	**<0.001**	**<0.001**
Distance traveled (m)	L	26.9 ± 2.0	30.5 ± 1.6	18.5 ± 1.8	9.653	**<0.001**	0.43	NT	0.503	**0.011**	**<0.001**
** Free exploration test**											
Latency to enter arena (s)	A	111.3 ± 24.0	155.1 ± 36.2	374.6 ± 103.4	4.203	**0.029**	0.29	SR	0.414	**0.023**	0.263
Time spent in arena (s)	A	159.7 ± 24.5	204.2 ± 33.2	138.0 ± 38.0	1.013	0.373	0.05	NT	0.582	0.888	0.364
Entries into arena (#)	L	19.9 ± 2.4	21.6 ± 2.7	14.4 ± 3.8	1.326	0.280	0.08	NT	0.926	0.432	0.278

*Behavioral data are given as untransformed means of the three groups (5-HTT +/+, 5-HTT +/−, 5-HTT −/−) ± standard error of the mean (SEM). Statistical information presented: main effects of genotype (LMM: *F*-ratio, *p*-value), effect sizes (η^2^_*p*_), transformation used (Trans; NT = not transformed, SR = square root transformation, Log = logarithmic transformation). A = anxiety-like behavior, L = exploratory locomotion, bold = *p* ≤ 0.05, bold italic = *p* ≤ 0.1.*

#### Anxiety-Like Behavior

**FIGURE 3:**
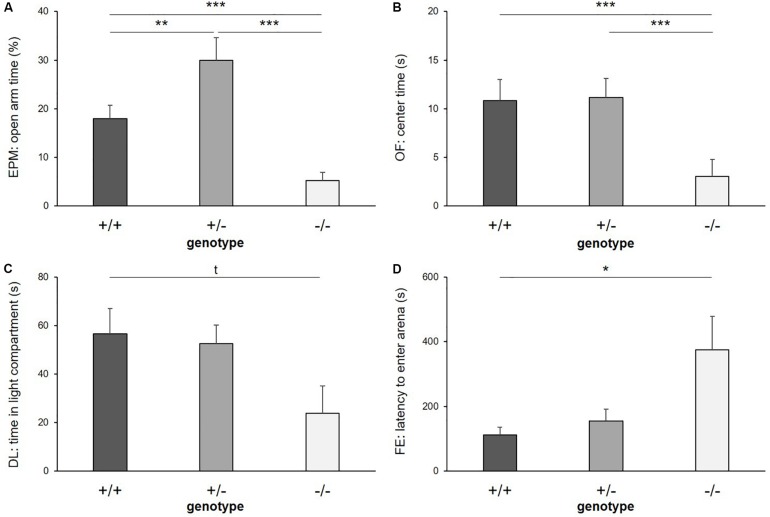
Anxiety-like behavior. **(A)** Time on open arms of Elevated plus maze, **(B)** time in center of Open field test, **(C)** time in light compartment of Dark light test, and **(D)** latency to enter arena of Free exploration test, displayed by 5-HTT +/+, 5-HTT +/−, and 5-HTT −/− mice. Data are presented as means ± SEM. Statistics: LMM; *post hoc* testing: Tukey’s test. Sample sizes: EPM, DL: *n*+/+ = 15, *n*+/− = 14, *n*−/− = 11. OF: *n*+/+ = 14, *n*+/− = 14, *n*−/− = 11. FE: *n*+/+ = 15, *n*+/− = 13 *n*−/− = 11. ^∗∗∗^*p* ≤ 0.001, ^∗∗^*p* ≤ 0.01, ^∗^*p* ≤ 0.05, *t* = *p* ≤ 0.1.

There was a significant main effect of genotype on anxiety-like behavior in the EPM, reflected by the time the mice spent on the open arms (*F*_(__2_,_39__)_ = 38.562, *p* ≤ 0.001; Figure 3A), the entries they made into the open arms (*F*_(__2_,_24__)_ = 8.404, *p* = 0.002) and the distance they traveled on the open arms of the apparatus (*F*_(__2_,_16__)_ = 25.384, *p* ≤ 0.001). *Post hoc* analysis showed that 5-HTT −/− mice displayed the highest levels of anxiety-like behavior, with the least time spent on the open arms (+/+ vs. −/−: *p* ≤ 0.001, +/− vs. −/−: *p* ≤ 0.001), the fewest entries made into these (+/+ vs. −/−: *p* = 0.023, +/− vs. −/−: *p* = 0.002) and the shortest distances traveled there (+/+ vs. −/−: *p* = 0.012, +/− vs. −/−: *p* ≤ 0.001). Interestingly, levels of anxiety-like behavior were lowest in the 5-HTT +/− group, indicated by significantly more time spent on the open arms (+/+ vs. +/−: *p* = 0.010) and longer distances traveled there compared to 5-HTT +/+ animals (+/+ vs. +/−: *p* = 0.002).

Furthermore, there was a main effect of genotype on anxiety-like behavior in the OF, indicated by the time the mice spent in the center (*F*_(__2_,_36__)_ = 11.380, *p* ≤ 0.001; Figure 3B) and the number of entries they made into the center of the apparatus (*F*_(__2_,_36__)_ = 14.710, *p* ≤ 0.001). Again, 5-HTT −/− mice displayed highest levels of anxiety-like behavior, as they spent less time in the center (+/+ vs. −/−: *p* ≤ 0.001, +/− vs. −/−: *p* ≤ 0.001) and entered it fewer times compared to both 5-HTT +/+ and 5-HTT +/− mice (+/+ vs. −/−: *p* ≤ 0.001, +/− vs. −/−: *p* ≤ 0.001).

Furthermore, there was a trend for a main effect of genotype on the time spent in the light compartment of the DL (*F*_(__2_,_32__)_ = 2.859, *p* = 0.072; Figure 3C). *Post hoc* comparisons revealed that by trend 5-HTT −/− mice spent less time in the light compartment compared to 5-HTT +/+ mice (+/+ vs. −/−: *p* = 0.073). However, we did not detect an effect of genotype on the latency to enter the light compartment (*F*_(__2_,_37__)_ = 0.851, *p* = 0.435).

In the FE, the latency to enter the arena was affected by genotype (*F*_(__2_,_21__)_ = 4.203, *p* = 0.029; Figure 3D), but we did not detect an effect of genotype on the time the animals spent there (*F*_(__2_,_36__)_ = 1.013, *p* = 0.373). Again, higher levels of anxiety-like behavior were found in 5-HTT −/− mice, indicated by longer latencies until they entered the arena compared to 5-HTT +/+ mice (+/+ vs. −/−: *p* = 0.023).

#### Exploratory Locomotion

**FIGURE 4:**
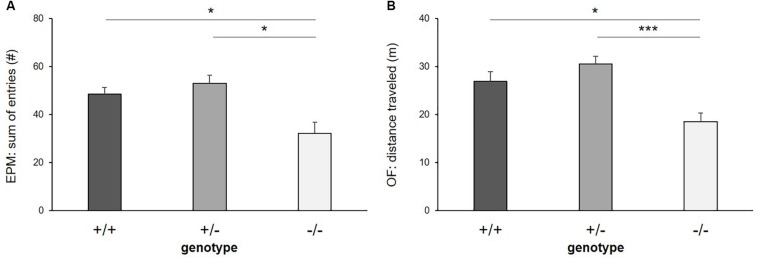
Exploratory locomotion. **(A)** Sum of entries into open and closed arms of Elevated plus maze, **(B)** distance traveled in Open field test. Data are given as means ± SEM. Statistics: LMM; *post hoc* testing: Tukey’s test. Sample sizes: EPM: *n*+/+ = 15, *n*+/− = 14, *n*−/− = 11. OF: *n*+/+ = 14, *n*+/− = 14, *n*−/− = 11. ^∗∗∗^*p* ≤ 0.001 and ^∗^*p* ≤ 0.05.

There was a significant main effect of genotype on exploratory locomotion in the EPM (sum of open and closed arm entries: *F*_(__2_,_16__)_ = 7.255, *p* = 0.006, Figure 4A) and in the OF (total distance traveled: *F*_(__2_,_26__)_ = 9.653, *p* ≤ 0.001, Figure 4B), however, not in the DL (entries into light compartment: *F*_(__2_,_33__)_ = 1.817, *p* = 0.178) and in the FE (entries into arena: *F*_(__2_,_30__)_ = 1.326, *p* = 0.280). *Post hoc* analysis showed that 5-HTT −/− mice displayed lower levels of exploratory locomotion compared to 5-HTT +/+ and 5-HTT +/− mice, as they made fewer entries into the open and closed arms of the EPM (+/+ vs. −/−: *p* = 0.013, +/− vs. −/−: *p* = 0.016) and traveled shorter distances in the OF (+/+ vs. −/−: *p* = 0.011, +/− vs. −/−: *p* ≤ 0.001).

## Discussion

In the present study, we aimed to investigate the role of judgment biases as potential endophenotypes for anxiety disorders in the 5-HTT mouse model. We therefore assessed the anxiety-like phenotype of mice varying in 5-HTT genotype in a battery of standardized tests, as well as their CBJ by using a touchscreen paradigm of potentially high translational value (Talpos and Steckler, 2013). While anxiety-like behavior was highest in 5-HTT −/− and lowest in 5-HTT +/− mice, we did not detect genotype differences in the animals’ judgment bias.

### Anxiety-Like Behavior

5-HTT −/− mice displayed lowest levels of exploratory locomotion and highest levels of anxiety-like behavior in the EPM, DL, and OF, reflecting the animals’ state anxiety. Anxiety-like behavior was also highest in these animals in the FE which targets trait anxiety. These results are consistent with previous findings and prove the applicability of the 5-HTT mouse model for the study of symptoms related to human anxiety disorders (Holmes et al., 2003; Carroll et al., 2007; Heiming et al., 2009).

Unexpectedly, 5-HTT +/− mice showed lowest levels of anxiety-like behavior in the EPM compared to wild type and homozygous knockout mice. In contrast to this, the majority of previous studies report heterozygous mice to be characterized by a tendency to show higher levels of anxiety-like behavior than wild type mice, similar to homozygous knockouts, yet, this anxiety-like phenotype only emerged in response to experimentally induced adversity (Carola et al., 2008; Jansen et al., 2010; van den Hove et al., 2011). Based on these studies, we would have expected heterozygous mice to rather show similar levels of anxiety-like behavior compared to wild type mice, as we did not provide any experimental treatment. In humans, however, reduced 5-HTT function is not only considered to confer a higher vulnerability to adversity, but rather a higher susceptibility to environmental influences in general, including also those of positive valence. This phenomenon has been referred to as the *differential susceptibility hypothesis* (Belsky et al., 2009). S-allele carriers are thus considered to be more plastic, instead of simply more vulnerable to adversity (Belsky et al., 2009). The differential susceptibility hypothesis is supported by first findings in mice: Kästner et al. (2015) showed heterozygous 5-HTT knockout mice to be more susceptible to the positive experience of cohabitation with a female. In comparison to wild type controls, heterozygous mice displayed a less-anxiety-like phenotype (Kästner et al., 2015).

The anxiety-like phenotype of the heterozygous mice in the present study matches the results of Kästner et al. (2015). However, we did not intentionally provide any beneficial experimental treatment. The only experimental procedure the mice underwent before entering the behavioral test battery was the cognitive bias task, including an intensive training phase. This might be a hint for a potential influence of the judgment bias paradigm itself on the behavioral profile of 5-HTT mice. A study addressing the effects of daily exposure to touchscreen training on mice supports this suggestion: Mallien et al. (2016) found mice to display elevated corticosterone levels in anticipation of touchscreen training. Such a glucocorticoid response does not only follow aversive conditions, but can also occur under conditions generally assumed to be of positive valence, e.g., during sexual behavior or environmentally enriched housing conditions (Marashi et al., 2003; Koolhaas et al., 2011). In accordance, Mallien et al. (2016) interpret their findings as a potential indicator of an enrichment-like effect elicited by the training procedure. Assuming a potentially beneficial effect of touchscreen training, our results might be another hint for a differential susceptibility conferred by reduced 5-HTT function.

### Cognitive Judgment Bias

There was a significant effect of condition in the CBJ test, showing that mice interpreted the five conditions differently. Scores resulted in a response curve that is typical for judgment bias tests, decreasing from the positive to the negative condition (e.g., Hintze et al., 2018). With this, we replicate previous results, supporting the assumption that the animals interpret the ambiguous cues with reference to the trained cues (Hintze et al., 2018; Krakenberg et al., 2019). This provides an argument in favor of choice scores as an applicable measure of judgment bias, adding support to a number of previous studies, e.g., in rats, relying on similar judgment bias indices (e.g., Papciak et al., 2013; Rafa et al., 2016). However, it is possible that confounding factors, e.g., perceptual biases on stimulus generalization, might skew the point of ambiguity and thus may affect responses to ambiguous conditions. Thus, the negative average choice scores in response to the middle condition cannot be interpreted as being solely due to a pessimistic-like judgment bias. Likewise, the asymmetric response pattern, i.e., average choice scores of about 0.9 in the positive, but only about −0.6 in the negative condition, could confound ambiguous cue interpretations. This should be taken into account when interpreting choice scores as measures of judgment bias. Interestingly, the NP and NN cues were not interpreted significantly differently than the trained reference cues. Similar findings have been made previously (e.g., Bateson and Matheson, 2007; Enkel et al., 2010; Hintze et al., 2018). It might be assumed that the NP and NN cues were not truly ambiguous to the animals. Shifting them closer toward the middle might be a useful step for future studies in order to enhance their degree of ambiguity. Moreover, it should be noted that this was the first time this novel paradigm has been used for assessing potential group differences in the judgment bias of mice. More studies are needed to further validate the task, e.g., via pharmacological or environmental manipulations, as it has been done for existing tasks already (e.g., Harding et al., 2004; Rygula et al., 2015).

We did not detect a significant effect of genotype on choice scores in the CBJ test, a finding of particular interest in the light of the differences we found in the animals’ anxiety-like behavior. This contradicts our expectations based on previous studies, in humans as well as in mice. In humans, 5-HTT genotype has been associated with biases in attention as well as ambiguous cue interpretation (Beevers et al., 2007; Fox et al., 2009; Fox and Standage, 2012). Despite the many physiological and behavioral similarities between human s-allele carriers and 5-HTT knockout mice, however, human studies hardly allow controlling for the background genotype or environmental effects across the subjects’ life span (Holmes et al., 2003). This should be kept in mind when comparing results between human and mouse studies, particularly because the 5-HTT s-allele is considered to confer a higher susceptibility to environmental influences (Belsky et al., 2009). Moreover, most studies in humans focused on attention biases, while here we assessed judgment bias (e.g., Beevers et al., 2007; Fox et al., 2009). It would thus be of great interest to additionally assess different subtypes of cognitive biases (attention, memory biases) in mice in the future, as these might be affected differently by 5-HTT function. Yet, to date, the necessary paradigms for assessing attention or memory bias in mice are lacking.

In mice, Kloke et al. (2014) found a very first hint for a role of 5-HTT genotype in the modulation of judgment biases, as they report a trend for homozygous 5-HTT knockout mice to display a pessimistic-like judgment bias. However, this finding was based on a very small sample size and needs further confirmation. Besides this, differences in their judgment bias paradigms and the one used here might have influenced test outcomes. While Kloke and co-workers applied a spatial discrimination task using a 3-arm maze, we made use of an automated touchscreen paradigm in the present study. The touchscreen method differs considerably from the maze task, e.g., in terms of experimenter influence, which is minimized in the touchscreen task due to less – and if necessary – cup handling (Hurst and West, 2010; Horner et al., 2013). Furthermore, the type of reinforcement used (high/low reward vs. reward/punishment) differed between the studies, and training duration and task complexity were increased in the touchscreen task. Such differences are considered to be capable of influencing test outcomes (Roelofs et al., 2016).

Apart from this, cognitive bias studies focusing on experimentally induced affective states do not yield consistent results either. It is true that the majority reports mood-congruent judgment biases, meaning that anxiety- and depression-like states mostly co-occur with a comparably pessimistic-like interpretation bias, while optimistic-like biases emerge for instance after providing environmental enrichment (e.g., Brydges et al., 2011; Salmeto et al., 2011; Richter et al., 2012; Papciak et al., 2013). Still, measures of anxiety-like behavior and judgment bias do not always reflect the same emotional valence. For instance, rats having experienced juvenile stress displayed higher levels of anxiety-like behavior and at the same time an optimistic-like judgment bias (Brydges et al., 2012). Likewise, a study in sheep reports an optimistic-like judgment bias to co-occur with higher levels of anxiety-like behavior (Destrez et al., 2014), and a study in hamsters found treatment-dependent differences in judgment bias, but not in anxiety-like behavior (Bethell and Koyama, 2015).

Taken together, evidence from previous studies as well as the findings presented here indicate that there is no straightforward association between 5-HTT genotype and judgment bias. From a genetic perspective, this finding is not surprising, since multiple genes are considered to be implicated in the modulation of such complex behavioral traits (Plomin et al., 1994; Canli and Lesch, 2007). Adding to this, in humans, optimism and pessimism are considered to be heritable to an estimate of only about 25% (Plomin et al., 1992). This emphasizes the role of environmental factors which, often in interaction with a genetic predisposition, constitute important modulators of behavioral traits (Caspi and Moffitt, 2006). More research is needed to gain further insights into the function of cognitive biases as potential endophenotypes for psychopathology.

## Data Availability Statement

The datasets generated for this study are available on request to the corresponding author.

## Ethics Statement

All procedures were reviewed and approved by both the Gesundheits-und Veterinäramt Münster, Nordrhein-Westfalen, Germany and the Landesamt für Natur, Umwelt und Verbraucherschutz Nordrhein-Westfalen, Recklinghausen, Germany.

## Author Contributions

SR, NS, and SK conceived the study. SR, NS, SK, and VK designed the experiments. VK carried out the experiments. VK and VvK conducted the statistical analysis of the data. SR and NS supervised the project. VK wrote the initial draft of the manuscript. SR, NS, SK, and VvK revised the manuscript critically for important intellectual content.

## Conflict of Interest

The authors declare that the research was conducted in the absence of any commercial or financial relationships that could be construed as a potential conflict of interest.

## Footnotes

1 http://www.r-project.org

## References

[B1] Archer J. (1973). Tests for emotionality in rats and mice: a review. *Anim. Behav.* 21 205–235. 10.1016/s0003-3472(73)80065-x 4578750

[B2] Bandelow B. Michaelis S. M. (2015). Epidemiology of anxiety disorders in the 21st century. *Dialog. Clin. Neurosci.* 17 327–335. 26487813 10.31887/DCNS.2015.17.3/bbandelow PMC4610617

[B3] Bates D. Maechler M. Bolker B. Walker S. (2015). Fitting linear mixed-effects models using lme4. *J. Statist. Softw.* 67 1–48. 10.18637/jss.v067.i01

[B4] Bateson M. Matheson S. M. (2007). Performance on a categorisation task suggests that removal of environmental enrichment inducespessimism’in captive European starlings (Sturnus vulgaris). *Anim. Welf.* 16 33–36.

[B5] Beevers C. G. Gibb B. E. McGeary J. E. Miller I. W. (2007). Serotonin transporter genetic variation and biased attention for emotional word stimuli among psychiatric inpatients. *J. Abnorm. Psychol.* 116 208–212. 10.1037/0021-843X.116.1.208 17324031

[B6] Belsky J. Jonassaint C. Pluess M. Stanton M. Brummett B. Williams R. (2009). Vulnerability genes or plasticity genes? *Mol. Psychiatr.* 14 746–754. 10.1038/mp.2009.44 19455150 PMC2834322

[B7] Bengel D. Murphy D. L. Andrews A. M. Wichems C. H. Feltner D. Heils A. (1998). Altered brain serotonin homeostasis and locomotorinsensitivity to 3,4-Methylenedioxymethamphetamine (“Ecstasy”) in serotonin transporter-deficient mice. *Mol. Pharmacol.* 53 649–655. 10.1124/mol.53.4.649 9547354

[B8] Bethell E. J. (2015). A “How-To” guide for designing judgment bias studies to assess captive animal welfare. *J. Appl. Anim. Welf. Sci.* 18 18–42. 10.1080/10888705.2015.1075833 26440495

[B9] Bethell E. J. Koyama N. F. (2015). Happy hamsters? enrichment induces positive judgement bias for mildly (but not truly) ambiguous cues to reward and punishment in *Mesocricetus auratus*. *R. Soc. Open Sci.* 2:140399. 10.1098/rsos.140399 26587255 PMC4632568

[B10] Brydges N. M. Hall L. Nicolson R. Holmes M. C. Hall J. (2012). The effects of juvenile stress on anxiety, cognitive bias and decision making in adulthood: a rat model. *PLoS One* 7:e48143. 10.1371/journal.pone.0048143 23118942 PMC3485359

[B11] Brydges N. M. Leach M. Nicol K. Wright R. Bateson M. (2011). Environmental enrichment induces optimistic cognitive bias in rats. *Anim. Behav.* 81 169–175. 10.1016/j.anbehav.2010.09.030

[B12] Burman O. H. P. Parker R. M. A. Paul E. S. Mendl M. T. (2009). Anxiety-induced cognitive bias in non-human animals. *Physiol. Behav.* 98 345–350. 10.1016/j.physbeh.2009.06.012 19560479

[B13] Bussey T. J. Holmes A. Lyon L. Mar A. C. McAllister K. A. L. Nithianantharajah J. (2012). New translational assays for preclinical modelling of cognition in schizophrenia: the touchscreen testing method for mice and rats. *Neuropharmacology* 62 1191–1203. 10.1016/j.neuropharm.2011.04.011 21530550 PMC3168710

[B14] Canli T. Lesch K.-P. (2007). Long story short: the serotonin transporter in emotion regulation and social cognition. *Nat. Neurosci.* 10 1103–1109. 10.1038/nn1964 17726476

[B15] Carola V. Frazzetto G. Pascucci T. Audero E. Puglisi-Allegra S. Cabib S. (2008). Identifying molecular substrates in a mouse model of the serotonin transporter x environment risk factor for anxiety and depression. *Biol. Psychiatry* 63 840–846. 10.1016/j.biopsych.2007.08.013 17949690

[B16] Carroll J. C. Boyce-Rustay J. M. Millstein R. Yang R. Wiedholz L. M. Murphy D. L. (2007). Effects of mild early life stress on abnormal emotion-related behaviors in 5-HTT knockout mice. *Behav. Genet.* 37 214–222. 10.1007/s10519-006-9129-9 17177116

[B17] Caspi A. Hariri A. R. Holmes A. Uher R. Moffitt T. E. (2010). Genetic sensitivity to the environment: the case of the serotonin transporter gene and its implications for studying complex diseases and traits. *Am. J. Psychiatr.* 167 509–527. 10.1176/appi.ajp.2010.09101452 20231323 PMC2943341

[B18] Caspi A. Moffitt T. E. (2006). Gene–environment interactions in psychiatry: joining forces with neuroscience. *Nat. Rev. Neurosci.* 7 583–590. 10.1038/nrn1925 16791147

[B19] Caspi A. Sugden K. Moffitt T. E. Taylor A. Craig I. W. Harrington H. (2003). Influence of life stress on depression: moderation by a polymorphism in the 5-HTT gene. *Science* 301 386–389. 10.1126/science.1083968 12869766

[B20] Crawley J. N. Goodwin F. K. (1980). Preliminary report of a simple animal behavior model for the anxiolytic effects of benzodiazepines. *Pharmacol. Biochem. Behav.* 13 167–170. 10.1016/0091-3057(80)90067-2 6106204

[B21] Destrez A. Deiss V. Leterrier C. Calandreau L. Boissy A. (2014). Repeated exposure to positive events induces optimistic-like judgment and enhances fearfulness in chronically stressed sheep. *Appl. Anim. Behav. Sci.* 154 30–38. 10.1016/j.applanim.2014.01.005

[B22] Enkel T. Gholizadeh D. von Bohlen Und Halbach O. Sanchis-Segura C. Hurlemann R. Spanagel R. (2010). Ambiguous-cue interpretation is biased under stress- and depression-like states in rats. *Neuropsychopharmacology* 35 1008–1015. 10.1038/npp.2009.204 20043002 PMC3055368

[B23] Fox E. Ridgewell A. Ashwin C. (2009). Looking on the bright side: biased attention and the human serotonin transporter gene. *Proc. Biol. Sci.* 276 1747–1751. 10.1098/rspb.2008.1788 19324793 PMC2674488

[B24] Fox E. Standage H. (2012). Variation on the serotonin transporter gene and bias in the interpretation of ambiguity. *J. Cogn. Psychol.* 24 106–114. 10.1080/20445911.2011.613821

[B25] Greenberg B. D. Li Q. Lucas F. R. Hu S. Sirota L. A. Benjamin J. (2000). Association between the serotonin transporter promoter polymorphism and personality traits in a primarily female population sample. *Am. J. Med. Genet.* 96 202–216. 10.1002/(sici)1096-8628(20000403)96:2<202::aid-ajmg16>3.0.co;2-j 10893498

[B26] Griebel G. Belzung C. Misslin R. Vogel E. (1993). The free-exploratory paradigm: an effective method for neophobic behaviour in mice and testing potential neophobia-reducing drugs. *Behav. Pharmacol.* 4 637–644. 11224232

[B27] Gross C. Hen R. (2004). The developmental origins of anxiety. *Nat. Rev. Neurosci.* 5 545–552. 10.1038/nrn1429 15208696

[B28] Harding E. J. Paul E. S. Mendl M. (2004). Cognitive bias and affective state. *Nature* 427:6972. 10.1038/427312a 14737158

[B29] Heiming R. S. Jansen F. Lewejohann L. Kaiser S. Schmitt A. Lesch K. P. (2009). Living in a dangerous world: the shaping of behavioral profile by early environment and 5-HTT genotype. *Front. Behav. Neurosci.* 3:26. 10.3389/neuro.08.026.2009 19826611 PMC2759357

[B30] Hintze S. Melotti L. Colosio S. Bailoo J. D. Boada-Saña M. Würbel H. (2018). A cross-species judgement bias task: integrating active trial initiation into a spatial Go/No-go task. *Sci. Rep.* 8:5104. 10.1038/s41598-018-23459-3 29572529 PMC5865189

[B31] Holmes A. Yang R. J. Lesch K.-P. Crawley J. N. Murphy D. L. (2003). Mice lacking the serotonin transporter exhibit 5-HT(1A) receptor-mediated abnormalities in tests for anxiety-like behavior. *Neuropsychopharmacology* 28 2077–2088. 10.1038/sj.npp.1300266 12968128

[B32] Horner A. E. Heath C. J. Hvoslef-Eide M. Kent B. A. Kim C. H. Nilsson S. R. O. (2013). The touchscreen operant platform for testing learning and memory in rats and mice. *Nat. Protoc.* 8 1961–1984. 10.1038/nprot.2013.122 24051959 PMC3914026

[B33] Hurst J. L. West R. S. (2010). Taming anxiety in laboratory mice. *Nat. Methods* 7 825–826. 10.1038/nmeth.1500 20835246

[B34] Jansen F. Heiming R. S. Lewejohann L. Touma C. Palme R. Schmitt A. (2010). Modulation of behavioural profile and stress response by 5-HTT genotype and social experience in adulthood. *Behav. Brain Res.* 207 21–29. 10.1016/j.bbr.2009.09.033 19782704

[B35] Jones S. Neville V. Higgs L. Paul E. S. Dayan P. Robinson E. S. J. (2018). Assessing animal affect. An automated and self-initiated judgement bias task based on natural investigative behaviour. *Sci. Rep.* 8:116. 10.1038/s41598-018-30571-x 30120315 PMC6098098

[B36] Kästner N. Richter S. H. Lesch K.-P. Schreiber R. S. Kaiser S. Sachser N. (2015). Benefits of a “vulnerability gene”? A study in serotonin transporter knockout mice. *Behav. Brain Res.* 283 116–120. 10.1016/j.bbr.2015.01.031 25629942

[B37] Kloke V. Schreiber R. S. Bodden C. Möllers J. Ruhmann H. Kaiser S. (2014). Hope for the best or prepare for the worst? Towards a spatial cognitive bias test for mice. *PLoS One* 9:e105431. 10.1371/journal.pone.0105431 25137069 PMC4138164

[B38] Koolhaas J. M. Bartolomucci A. Buwalda B. Boer S. F. de Flügge G. Korte S. M. (2011). Stress revisited: a critical evaluation of the stress concept. *Neurosci. Biobehav. Rev.* 35 1291–1301. 10.1016/j.neubiorev.2011.02.003 21316391

[B39] Krakenberg V. Woigk I. Garcia Rodriguez L. Kästner N. Kaiser S. Sachser N. (2019). Technology or ecology? New tools to assess cognitive judgement bias in mice. *Behav. Brain Res.* 362 279–287. 10.1016/j.bbr.2019.01.021 30654122

[B40] Kuznetsova A. Brockhoff P. B. Christensen R. H. B. (2017). lmerTest package: tests in linear mixed effects models. *J. Statist. Softw.* 82 1–26. 10.18637/jss.v082.i13

[B41] Lakens D. (2013). Calculating and reporting effect sizes to facilitate cumulative science: a practical primer for t-tests and ANOVAs. *Front. Psychol.* 4:863. 10.3389/fpsyg.2013.00863 24324449 PMC3840331

[B42] Lenth R. V. (2016). Least-squares means: the r package lsmeans. *J. Statist. Softw.* 69 1–33. 10.18637/jss.v069.i01

[B43] Lesch K.-P. Bengel D. Heils A. Sabol S. Z. Greenberg B. D. Petri S. (1996). Association of anxiety-related traits with a polymorphism in the serotonin transporter gene regulatory region. *Science* 274 1527–1531. 10.1126/science.274.5292.1527 8929413

[B44] Lister R. G. (1987). The use of a plus-maze to measure anxiety in the mouse. *Psychopharmacology* 92 180–185. 3110839 10.1007/BF00177912

[B45] Lister R. G. (1990). Ethologically-based animal models of anxiety disorders. *Pharmacol. Ther.* 46 321–340. 10.1016/0163-7258(90)90021-s 2188266

[B46] Mallien A. S. Palme R. Richetto J. Muzzillo C. Richter S. H. Vogt M. A. (2016). Daily exposure to a touchscreen-paradigm and associated food restriction evokes an increase in adrenocortical and neural activity in mice. *Horm. Behav.* 81 97–105. 10.1016/j.yhbeh.2016.03.009 27059527

[B47] Marashi V. Barnekow A. Ossendorf E. Sachser N. (2003). Effects of different forms of environmental enrichment on behavioral, endocrinological, and immunological parameters in male mice. *Horm. Behav.* 43 281–292. 10.1016/S0018-506X(03)00002-3 12694638

[B48] Mathews A. MacLeod C. (1994). Cognitive approaches to emotion and emotional disorders. *Annu. Rev. Psychol.* 45 25–50. 10.1146/annurev.psych.45.1.25 8135504

[B49] Mathews A. MacLeod C. (2005). Cognitive vulnerability to emotional disorders. *Ann. Rev. Clin. Psychol.* 1 167–195. 10.1146/annurev.clinpsy.1.102803.143916 17716086

[B50] Mazzanti C. M. Lappalainen J. Long J. C. Bengel D. Naukkarinen H. Eggert M. (1998). Role of the serotonin transporter promoter polymorphism in anxiety-related traits. *Arch. Gen. Psychiatry* 55 936–940. 10.1001/archpsyc.55.10.936 9783565

[B51] Murphy D. L. Li Q. Engel S. Wichems C. H. Andrews A. M. Lesch K.-P. (2001). Genetic perspectives on the serotonin transporter. *Brain Res. Bull.* 56 487–494. 10.1016/s0361-9230(01)00622-0 11750794

[B52] Papciak J. Popik P. Fuchs E. Rygula R. (2013). Chronic psychosocial stress makes rats more ‘pessimistic’ in the ambiguous-cue interpretation paradigm. *Behav. Brain Res.* 256 305–310. 10.1016/j.bbr.2013.08.036 23993861

[B53] Pellow S. Chopin P. File S. E. Briley M. (1985). Validation of open : closed arm entries in an elevated plus-maze as a measure of anxiety n the rat. *J. Neurosci. Methods* 14 149–167. 10.1016/0165-0270(85)90031-7 2864480

[B54] Plomin R. Owen M. J. McGuffin P. (1994). The genetic basis of complex human behaviors. *Science* 264 1733–1739. 10.1126/science.8209254 8209254

[B55] Plomin R. Scheier M. F. Bergman C. S. Pedersen N. L. Nesselroade J. R. McClearn G. E. (1992). Optimism, pessimism and mental health: a twin/adoption analysis. *Pers. Individ. Differ.* 13 921–930. 10.1016/0191-8869(92)90009-E

[B56] Rafa D. Kregiel J. Popik P. Rygula R. (2016). Effects of optimism on gambling in the rat slot machine task. *Behav. Brain Res.* 300 97–105. 10.1016/j.bbr.2015.12.013 26698397

[B57] Ramos A. Mormède P. (1998). Stress and emotionality: a multidimensional and genetic approach. *Neurosci. Biobehav. Rev.* 22 33–57. 10.1016/s0149-7634(97)00001-8 9491939

[B58] Richter S. H. Schick A. Hoyer C. Lankisch K. Gass P. Vollmayr B. (2012). A glass full of optimism: enrichment effects on cognitive bias in a rat model of depression. *Cogn. Affect. Behav. Neurosci.* 12 527–542. 10.3758/s13415-012-0101-2 22644760

[B59] Richter S. H. Vogel A. S. Ueltzhöffer K. Muzzillo C. Vogt M. A. Lankisch K. (2014). Touchscreen-paradigm for mice reveals cross-species evidence for an antagonistic relationship of cognitive flexibility and stability. *Front. Behav. Neurosci.* 8:154. 10.3389/fnbeh.2014.00154 24834036 PMC4017158

[B60] Roelofs S. Boleij H. Nordquist R. E. van der Staay F. J. (2016). Making decisions under ambiguity: judgment bias tasks for assessing emotional state in animals. *Front. Behav. Neurosci.* 10:119. 10.3389/fnbeh.2016.00119 27375454 PMC4899464

[B61] Rygula R. Golebiowska J. Kregiel J. Holuj M. Popik P. (2015). Acute administration of lithium, but not valproate, modulates cognitive judgment bias in rats. *Psychopharmacology* 232 2149–2156. 10.1007/s00213-014-3847-0 25537337 PMC4432082

[B62] Rygula R. Papciak J. Popik P. (2013). Trait pessimism predicts vulnerability to stress-induced anhedonia in rats. *Neuropsychopharmacology* 38 2188–2196. 10.1038/npp.2013.116 23660704 PMC3773668

[B63] Salmeto A. L. Hymel K. A. Carpenter E. C. Brilot B. O. Bateson M. Sufka K. J. (2011). Cognitive bias in the chick anxiety-depression model. *Brain Res.* 1373 124–130. 10.1016/j.brainres.2010.12.007 21156165

[B64] Talpos J. Steckler T. (2013). Touching on translation. *Cell Tissue Res.* 354 297–308. 10.1007/s00441-013-1694-7 23949375

[B65] Treit D. Fundytus M. (1989). Thigmotaxis as a test for anxiolytic activity in rats. *Pharmacol. Biochem. Behav.* 31 959–962. 10.1016/0091-3057(88)90413-3 3252289

[B66] van den Hove D. Jakob S. B. Schraut K.-G. Kenis G. Schmitt A. G. Kneitz S. (2011). Differential effects of prenatal stress in 5-Htt deficient mice: towards molecular mechanisms of gene × environment interactions. *PLoS One* 6:e22715. 10.1371/journal.pone.0022715 21857948 PMC3155516

