# Supplementary material for: Differential Effects of Serotonin Transporter Genotype on Anxiety-Like Behavior and Cognitive Judgment Bias in Mice
Source: Front Behav Neurosci. 2019 Dec 3;13:263. doi: 10.3389/fnbeh.2019.00263 (PMC6902087; doi:10.3389/fnbeh.2019.00263)
Supplement: Supplementary file 1 [file Data_Sheet_1.docx]

Supplementary Material

# Supplementary Tables

Supplementary table 1: Statistical analysis of cognitive judgment bias test. Data are given as untransformed means of the three groups (5-HTT +/+, 5-HTT +/-, 5-HTT -/-) ± standard error of the mean (SEM). Statistical information presented: main effects of genotype, condition, and genotype x condition interaction (F-ratio, p-value), effect sizes (η^2^_p_).

|  | **5-HTT +/+** | | | **5-HTT +/-** | | | **5-HTT -/-** | | | **Post hoc comparisons** | |
| --- | --- | --- | --- | --- | --- | --- | --- | --- | --- | --- | --- |
|  | **Mean** | **±** | **SEM** | **Mean** | **±** | **SEM** | **Mean** | **±** | **SEM** | **Pair** | **p** |
| Positive | 0.89 | ± | 0.03 | 0.89 | ± | 0.02 | 0.88 | ± | 0.04 | P - NP | 0.9852 |
| Near positive | 0.88 | ± | 0.05 | 0.84 | ± | 0.05 | 0.87 | ± | 0.04 | P- M | < 0.001 |
| Middle | -0.09 | ± | 0.07 | -0.11 | ± | 0.09 | -0.20 | ± | 0.09 | P- NN | < 0.001 |
| Near negative | -0.55 | ± | 0.07 | -0.53 | ± | 0.07 | -0.56 | ± | 0.08 | P - N | < 0.001 |
| Negative | -0.65 | ± | 0.04 | -0.56 | ± | 0.05 | -0.58 | ± | 0.05 | NP - M | < 0.001 |
|  | **ANOVA** | | | | | | | | | P - NN | < 0.001 |
|  | **F** | | | **p** | | | **η^2^_p_** | | | NP - N | < 0.001 |
| Genotype | 0.1 | | | 0.91 | | | < 0.001 | | | M - NN | < 0.001 |
| Condition | 501.73 | | | **< 0.001** | | | 0.93 | | | M - N | < 0.001 |
| Interaction | 0.46 | | | 0.88 | | | 0.02 | | | NN - N | 0.8337 |

# Supplementary Figures


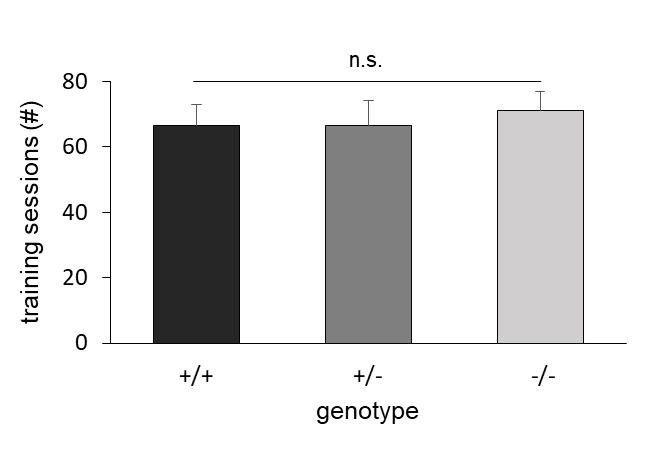


Supplementary figure 1: Training duration. Data are given as mean numbers of training sessions until reaching criterion for the three groups of mice (5-HTT +/+, 5-HTT +/-, 5-HTT -/-) ± standard error of the mean (SEM).Statistical analysis: IBM SPSS statistics, version 25. Kruskal-Wallis test, H = 0.751, p = 0.687. n.s.: not significant.


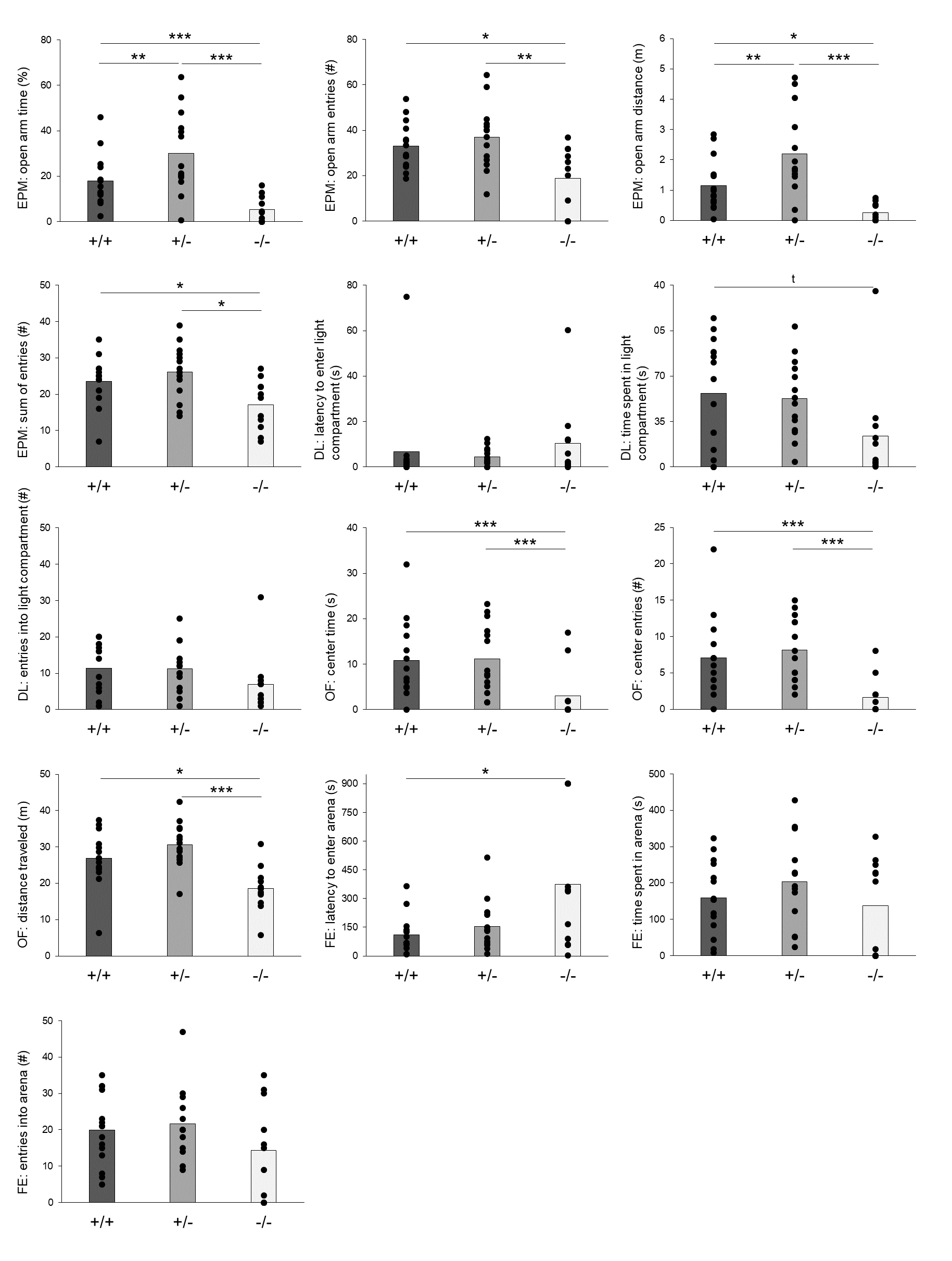


Supplementary figure 2: Anxiety-like and exploratory behavior. Data are given as bars displaying group means and individual values. X-axes with acronyms for genotypes: +/+: 5-HTT +/+ mice, +/-: 5-HTT +/- mice, -/-: 5-HTT -/- mice. t: 0.05 < p ≤ 0.09, *p ≤ 0.05, **p ≤ 0.01, ***p ≤ 0.001.


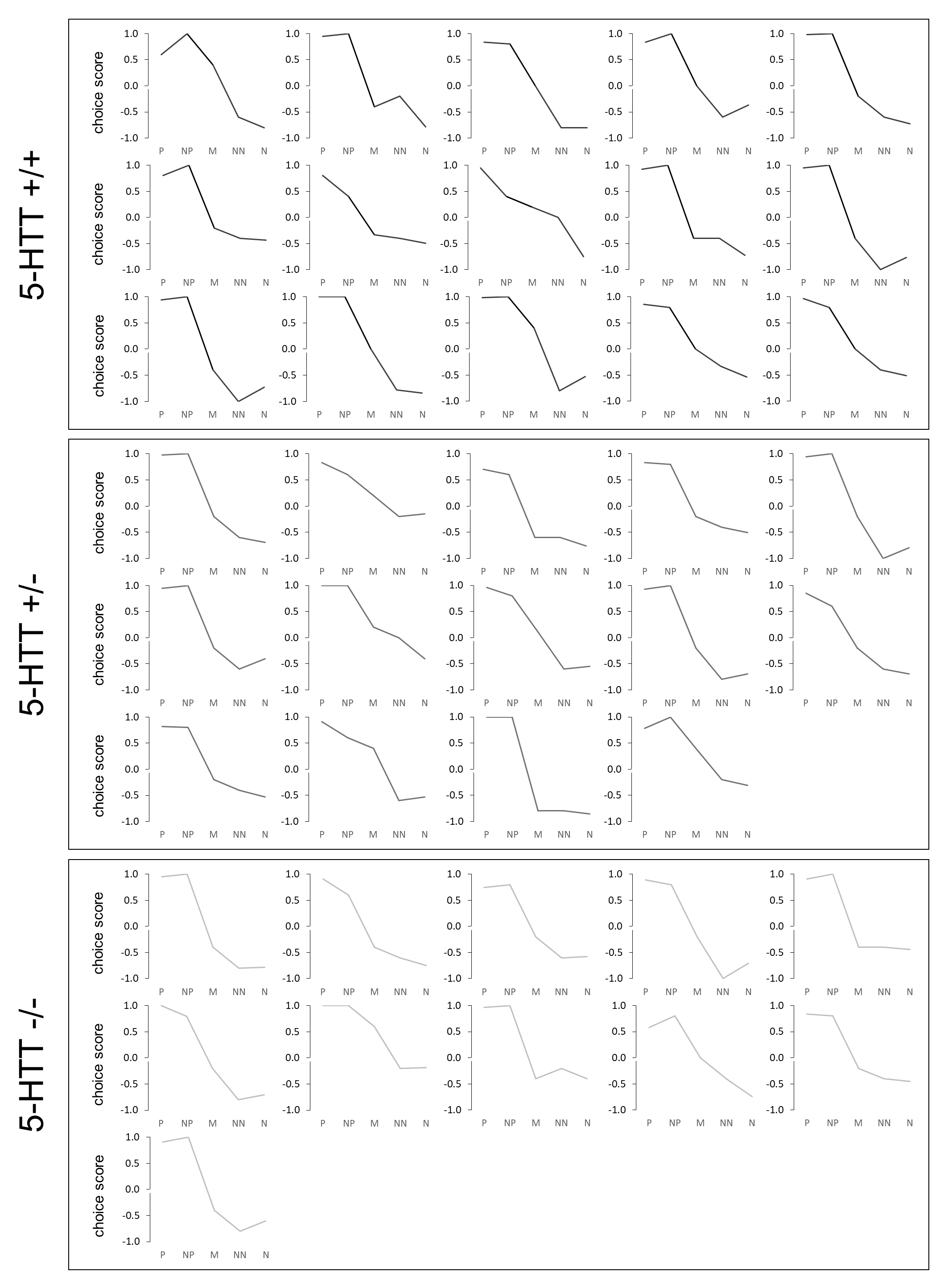


Supplementary figure 3: Individual choice scores of 5-HTT +/+, +/- and -/- mice. P: positive condition, NP: near positive condition, M: middle condition, NN: near negative condition, N: negative condition.

***
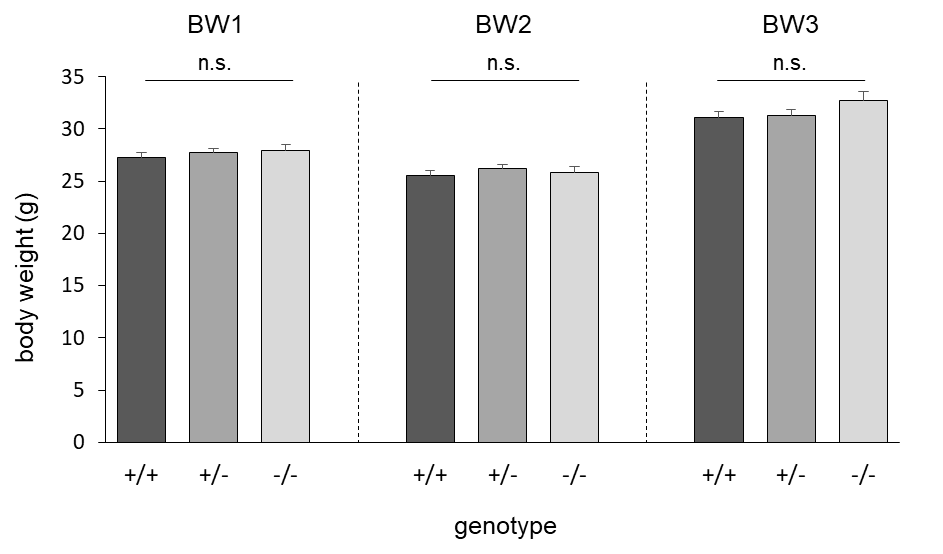
***

**Supplementary Figure 4: Body weights of 5-HTT +/+, +/- and -/- mice.** BW1: body weight before food restriction (*ad libitum* feeding), BW2: body weight at end of judgment bias test phase (food restriction phase), BW3: last body weight before behavioral test phase (*ad libitum* feeding). Bars represent means ± SEM. Statistical analysis: IBM SPSS statistics, version 25. ANOVA: BW1: F = 0.439, p = 0.648. BW2: F = 0.582, p = 0.564. BW3: F = 1.552, p = 0.225. n.s.: not significant.
